# Design of a Prospective Study on Pharmacokinetic-Guided Dosing of Prophylactic Factor Replacement in Hemophilia A and B (OPTI-CLOT TARGET Study)

**DOI:** 10.1055/a-1760-0105

**Published:** 2022-03-10

**Authors:** Tine M.H.J. Goedhart, Laura H. Bukkems, Michiel Coppens, Karin J. Fijnvandraat, Saskia E.M. Schols, Roger E.G. Schutgens, Jeroen Eikenboom, Floor C.J.I. Heubel-Moenen, Paula F. Ypma, L. Nieuwenhuizen, K. Meijer, Frank W. G. Leebeek, Ron A.A. Mathôt, Marjon H. Cnossen

**Affiliations:** 1 Department of Pediatric Hematology and Oncology, Erasmus MC Sophia Children's Hospital, University Medical Center Rotterdam, Rotterdam, The Netherlands; 2 Department of Clinical Pharmacology - Hospital Pharmacy, Amsterdam UMC, University of Amsterdam, Amsterdam, The Netherlands; 3 Department of Vascular Medicine, Amsterdam Cardiovascular Sciences, Amsterdam UMC, University of Amsterdam, Amsterdam, The Netherlands; 4 Department of Pediatric Hematology, Amsterdam UMC, Emma Children's Hospital, University of Amsterdam, Meibergdreef 9, Amsterdam, the Netherlands; 5 Department of Hematology, Radboud University Medical Center, Nijmegen, and the Hemophilia Treatment Center Nijmegen-Eindhoven-Maastricht, The Netherlands; 6 Van Creveldkliniek, University Medical Center Utrecht, Utrecht, The Netherlands; 7 Department of Internal Medicine, Division of Thrombosis and Hemostasis, Leiden University Medical Center, Leiden, The Netherlands; 8 Department of Hematology, Maastricht University Medical Center, Maastricht, The Netherlands; 9 Department of Hematology, Haga Hospital, The Hague, The Netherlands; 10 Department of Internal Medicine, Maxima Medical Center, Veldhoven, The Netherlands; 11 Department of Hematology, University Medical Center Groningen, Groningen, The Netherlands; 12 Department of Hematology, Erasmus MC, University Medical Center Rotterdam, Rotterdam, The Netherlands

**Keywords:** hemophilia, pharmacokinetics, factor VIII, factor IX, prophylaxis

## Abstract

In resource-rich countries, almost all severe hemophilia patients receive prophylactic replacement therapy with factor concentrates to prevent spontaneous bleeding in joints and muscles to decrease the development of arthropathy and risk of long-term disability. Pharmacokinetic (PK)-guided dosing can be applied to individualize factor replacement therapy, as interindividual differences in PK parameters influence factor VIII (FVIII) and FIX activity levels. PK-guided dosing may therefore lead to more optimal safeguarding of FVIII/FIX levels during prophylaxis and on demand treatment. The OPTI-CLOT TARGET study is a multicenter, nonrandomized, prospective cohort study that aims to investigate the reliability and feasibility of PK-guided prophylactic dosing of factor concentrates in hemophilia-A and -B patients in daily clinical practice. At least 50 patients of all ages on prophylactic treatment using standard half-life (SHL) and extended half-life (EHL) factor concentrates will be included during 9 months and will receive PK-guided treatment. As primary endpoint, a minimum of four FVIII/FIX levels will be compared with FVIII/FIX levels as predicted by Bayesian forecasting. Secondary endpoints are the association of FVIII and FIX levels with bleeding episodes and physical activity, expectations and experiences, economic analyses, and optimization of population PK models. This study will lead to more insight in the reliability and feasibility of PK-guided dosing in hemophilia patients. Moreover, it will contribute to personalization of treatment by greater knowledge of dosing regimens needed to prevent and treat bleeding in the individual patient and provide evidence to more clearly associate factor activity levels with bleeding risk.

## Introduction

Hemophilia A and hemophilia B are X-linked recessive bleeding disorders caused by a deficiency or dysfunction of coagulation factor VIII (FVIII) or FIX, respectively. Severe patients (FVIII/FIX < 0.01 IU/mL) and some moderate-to-severe patients (FVIII/FIX: 0.01–0.05 IU/mL) suffer from spontaneous bleeding or bleeding after minimal trauma. Prophylactic treatment by intravenous administration of factor concentrates aims to prevent (spontaneous) bleedings in joint and muscles and subsequent arthropathy with potential long-term disability. 1 2

During prophylaxis theoretically, FVIII/FIX trough levels is targeted to >0.01 IU/mL. This principle is based on observations by Ahlberg as early as in 1965 that bleeding phenotype and joint status are strikingly different between severe and moderate-to-severe hemophilia patients with only minimal baseline FVIII level differences (<0.01 vs. 0.01–0.05 IU/mL). 3 To achieve these FVIII/FIX trough levels during prophylaxis, FVIII/FIX concentrates are mostly prescribed according to body weight. 2 Remarkably, it is still not usual clinical practice to standardly measure and monitor trough FVIII/FIX levels when no bleeding occurs. To personalize dosing, information on trough FVIII/FIX levels is of value to establish if prophylaxis is adequate for each individual patient, during follow–up, and in varying circumstances and when dosing on demand. In addition, the lack of knowledge of achieved FVIII/FIX levels impedes proper switching to novel long-acting factor concentrates due to uncertainties which trough FVIII/FIX target levels should be targeted to prolongate earlier effective prophylactic treatment to prevent bleeding, especially in relation to physical activity or sports.

### Pharmacokinetic-Guided Dosing

Large interindividual variability exists in the pharmacokinetics (PKs) of FVIII/FIX concentrates as demonstrated by Björkman et al among others. 4 5 6 To understand and predict the consequences of the interindividual variability of factor concentrates in individuals, population PK models have been constructed for prophylaxis 4 7 8 9 10 11 12 13 14 15 16 17 18 19 20 21 22 23 24 25 26 27 28 29 30 31 32 and perioperative treatment 33 with standard half-life (SHL) and extended half-life (EHL) FVIII and FIX concentrates for hemophilia-A and -B patients, respectively. With these population PK models, Bayesian forecasting can be performed. Herewith, individual PK parameters are estimated which are subsequently used to calculate the adequate dose for an individual patient to achieve FVIII/FIX target levels, both trough and peak. The availability of population PK models has made limited sampling possible, making prior frequent blood sampling (>10 blood samples) 34 and a wash-out period redundant. PK-guided dosing has also been reported to not only be able to predict dosing requirements to attain certain target FVIII/FIX levels but also to decrease the amount of factor concentrates with concomitant reduction of costs. 33 Carlsson et al was the first to report a dose and cost reduction of 30% of FVIII concentrate without an increase in bleeding events, when PK-guided prophylactic dosing was compared with standard prophylactic dosing in a small patient sample. 35 However, a recent randomized controlled perioperative trial was not able to show a decrease in FVIII concentrate consumption, although achievement of FVIII target ranges was clearly more optimal. 36

We hypothesize PK-guided dosing leads to individualization of prophylaxis which is in accordance with the recommendations of the subcommittee on FVIII, FIX, and rare bleeding disorders of the International Society on Thrombosis and Haemostasis (ISTH). 37 PK-guided dosing may help achieve higher trough levels more efficiently when clinically indicated, as well as provide guidance when patients switch to alternative replacement factor concentrates, while taking cost and benefit of treatment into account. In addition, PK-guided dosing may lead to increased insight into the association between FVIII/FIX levels, bleeding (risk), and physical activity levels in individual patients as factor levels can be predicted at any time point and related to bleeding and activity. Therefore, we aim to prove that FVIII/FIX trough and peak levels as set by treating physician can be predicted and achieved reliably by application of PK-guided prophylaxis and that this intervention is feasible for patients and treatment teams.

## Objective

Investigate the reliability and feasibility of PK-guided prophylactic dosing of factor concentrates in hemophilia-A and -B patients in daily clinical practice.

## Methods

### Study Design

The OPTICLOT TARGET study is a multicenter, nonrandomized, prospective cohort study.

### Study Population

Patients will be recruited from the two Dutch Hemophilia treatment Centers; follows Erasmus MC, University Medical Center Rotterdam and Amsterdam University Medical Centers.

#### Inclusion Criteria

Inclusion criteria are as follows:

- Hemophilia-A and -B patients of all ages on prophylaxis.
- Prophylaxis with SHL or EHL factor concentrates.
- Written (parental) informed consent, according to local law and regulations.

#### Exclusion Criteria

Exclusion criteria are listed below:

- Patients with other severe congenital or acquired hemostatic abnormalities.
- General medical conditions which may interfere with participation in the study.
- Inability to adhere to prophylaxis and/or inability to keep detailed logs on infusion and bleeding episodes.
- Withdrawal of (parental) informed consent.
- Presence of FVIII/FIX inhibitor, leading to alternative treatment with bypassing products, immune toleration induction, and/or other immune modulating treatment.

### Outcome Measures

#### Primary Endpoints

Observed FVIII and FIX levels in comparison to FVIII and FIX levels are predicted by Bayesian forecasting. The predictive performance is deemed acceptable when at least 80% of the actual FVIII/FIX levels are within ± 25% of the predicted (target) values as stated by treating professional.

#### Secondary Endpoints

The four secondary endpoints are briefed below:

1. Association of (real world or predicted) FVIII/FIX levels with bleeding episodes and daily activities. Additionally, bleeds will be categorized according to the following subclassifications: total number of bleeds over time, number of spontaneous bleeds, number of traumatic bleeds, number of joint bleeds, number of target joint bleeds, and bleed severity.
2. Expectations, feasibility, and experience with PK-guided dosing with the different factor concentrates (SHL vs. EHL) as reported by patient/caretakers and physician will be measured using a visual analogue scale (VAS) questionnaire at the start and end of the study.
3. Economic analysis in which costs and benefits of standard prophylactic treatment and PK-guided prophylaxis are compared.
4. Analysis of described modifiers effecting PK parameters of FVIII/FIX concentrate to further optimize population PK models. Modifiers include demographics (such as lean body mass) and laboratory measurements (such as the von Willebrand Factor levels).

### Interventions

**Fig. 1:**
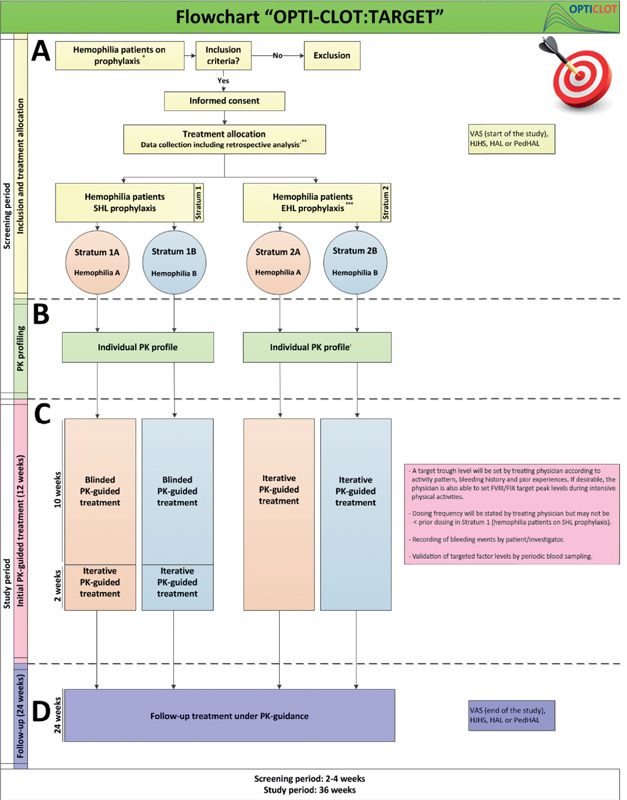
Flow chart. EHL, extended half-life; HAL, Hemophilia Activity List; HJHS, Hemophilia Joint Score; PedHAL, pediatric HAL; PK, pharmacokinetic(s); SHL, standard half-life; VAS, visual analogue scale *Non severe hemophilia patients will be analyzed separately. **In parallel with the prospective study, retrospective data analysis will be performed over a 12 month period prior to inclusion (if no PK profiling has been performed) or from PK profiling prior to inclusion. ***Patients in stratum 2 could undergo PK profiling during SHL prophylaxis as well as during EHL prophylaxis.

Study interventions are depicted in the flowchart ( Fig. 1 ). Patients will be categorized into strata according to type of hemophilia and type of factor concentrate (SHL or EHL). For all patients, the following patient characteristics and demographics will be collected: type of hemophilia, endogenous factor level, DNA mutation, age, height (cm), weight (kg), body mass index (BMI; kg/cm ^2^ ), lean body mass (kg), blood group, current other medication, and activity patterns.

Patients/caretakers will fill in the Hemophilia Activities List (HAL) and/or pediatric HAL (PedHAL) before initiation. Moreover, the Hemophilia Joint Health Score (HJHS) will be performed or must have been performed <12 months prior inclusion.

The validated PedHAL/HAL questionnaire and the HJHS are included as clinical parameters to systematically establish baseline values of functional outcome from the patient's perspective and to be informed of joint status, respectively. These clinical parameters may help to evaluate outcomes after implementation of PK guidance.

Furthermore, both patients/caretakers and the treating physician will fill in a specifically developed questionnaire using VAS scales, before the implementation of PK-guided dosing, considering the expectations with PK-guided dosing of prophylaxis. More specifically, in the questionnaire, questions are asked about satisfaction, being informed of factor levels, and expected burden of PK guidance. Moreover, when patients switch to an EHL factor concentrate, the reasons for switching are also asked.

**Fig. 2:**
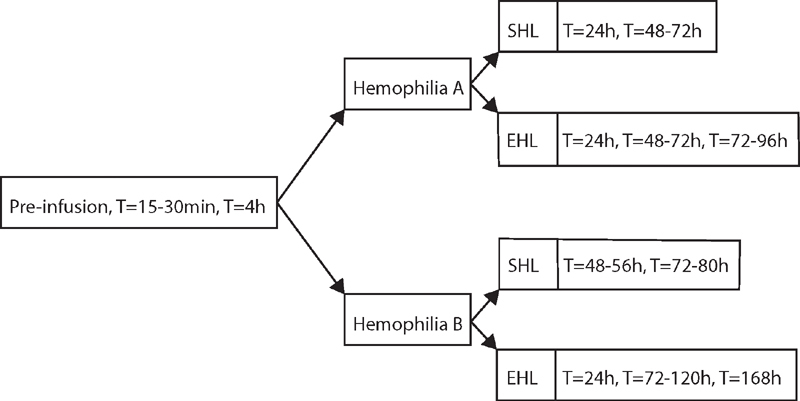
Time points (T) of laboratory tests during individual PK-profiling. A preinfusion, *t* = 15–30 minutes and t = 4 hours sample (left) are performed in all patients. The other time points (right) depend on hemophilia type and brand of factor concentrate. EHL, extended half-life; PK, pharmacokinetic; SHL, standard half-life.

An individual PK profile will be constructed after a factor concentrate dose of 35 to 50 IU/kg, depending on hemophilia type and age of the patient. The frequency and timing of blood sampling during PK profiling is depending on type of hemophilia and type of factor concentrate ( Fig. 2 ). No wash out period is required if three prior infusions and time points of infusion are documented. During sampling of the PK profile, laboratory tests will be performed according to Tables 1 and 2 .

**Table 1:** Laboratory tests during individual PK profiling for patients with hemophilia A

On FVIII-SHL	Preinfusion	T = 15–30 minute	T = 4 hours	T = 24 hours	T = 48–72 hours	
On FVIII-EHL	Preinfusion	T = 15–30 minute	T = 4 hours	T = 24 hours	T = 48 hours	T = 72–96 hours a
ASAT	X					
ALAT	X					
GGT	X					
LDH	X					
AF	X					
Albumin	X					
Urea	X					
Creatinine	X					
Hemoglobin	X					
Hematocrit	X					
Thrombocytes	X					
Blood group (if unknown)	X					
Factor VIII	X	X	X	X	X	X
VWF:Ag	X	X	X	X	X	X
VWF:Act	X	X	X	X	X	X
VWF:CB	X	X	X	X	X	X
VWFpp	X	X	X	X	X	X
Inhibitor FVIII (only ≥ 18 years)	X					
Bethesda FVIII	X					
Buffycoat	X					
APTT	X	X	X	X	X	X
PT/INR	X					
Factor V	X					
Fibrinogen	X					
Max of 10-mL citrate plasma	X	X	X	X	X	X

Abbreviations: AF, alkaline phosphatase; ALAT, alanine aminotransaminase; APTT, activated partial thromboplastin time; ASAT, aspartate aminotransferase; EHL, extended half-life; FIX, factor IX; FV, factor V; FVIII, factor VIII; GGT, gamma-glutamyltransferase; INR, international normalized ratio; LDH, lactate dehydrogenase; PFA, platelet function assay; PK, pharmacokinetic; PT, prothrombin time; SHL, standard half-life; T, time point; VWF:Act, von Willebrand's factor activity; VWF:Ag, von Willebrand factor antigen; VWF:CB, von Willebrand factor collagen binding; VWFpp, von Willebrand factor propeptide.

a Only in case of an EHL concentrate.

**Table 2:** Laboratory tests during individual PK profiling for patients with hemophilia B

On FIX-SHL	Preinfusion	T = 15–30 minutes	T = 4 hours	T = 48–56 hours	T = 72–80 hours	
On FIX-EHL	Preinfusion	T = 15–30 minutes	T = 4 hours	T = 24 hours	T = 72–120 hours	T = 168 hours a
ASAT	X					
ALAT	X					
GGT	X					
LDH	X					
AF	X					
Albumin	X					
Urea	X					
Creatinine	X					
Hemoglobin	X					
Hematocrit	X					
Thrombocytes	X					
Blood group (if unknown)	X					
Factor IX	X	X	X	X	X	X
Inhibitor FIX (only ≥ 18 years)	X					
Bethesda FIX	X					
Factor VIII	X					
VWF:Ag	X					
VWF:Act	X					
VWF:CB	X					
VWFpp	X					
Buffy coat	X					
APTT	X	X	X	X	X	X
PT + INR	X					
Factor V	X					
Fibrinogen	X					
Max of 10 mL citrate plasma	X	X	X	X	X	X

Abbreviations: AF, alkaline phosphatase; ALAT, alanine aminotransaminase; APTT, activated partial thromboplastin time; ASAT, aspartate aminotransferase; EHL, extended half-life; FIX, factor IX; FV, factor V; FVIII, factor VIII; GGT, gamma-glutamyltransferase; INR, international normalized ratio; LDH, lactate dehydrogenase; PFA, platelet function assay; PK, pharmacokinetic; PT, prothrombin time; SHL, standard half-life; T, time point; VWF:Act, von Willebrand factor activity; VWF:Ag, von Willebrand factor antigen; VWF:CB, von Willebrand factor collagen binding; VWFpp, von Willebrand factor propeptide.

a Only in case of an EHL concentrate.

**Table 3:** Laboratory tests during initial PK-guided treatment and follow-up

	Initial PK-guided treatment (12 weeks)			Follow-up treatment under PK guidance (24 weeks)	Bleeds (only when blood sampling is clinically indicated, during total study period)
Visit	1	2	3	1	additional
Hemophilia A					
FVIII	X	X	X	X	X
FIX					X
VWF:Ag					X
VWF:Act					X
VWF:CB					X
VWFpp					X
APTT	X	X	X	X	X
Inhibitor FVIII	X			X	(X)
Max of 10-mL citrate plasma	X	X	X	X	X
Hemophilia B					
FIX	X	X	X	X	X
FVIII					X
VWF:Ag					X
VWF:Act					X
VWF:CB					X
VWFpp					X
APTT	X	X	X	X	X
Inhibitor FIX			X	X	(X)
Max of 10-mL citrate plasma	X	X	X	X	X

Abbreviations: APTT, activated partial thromboplastin time; FIX, factor IX; FVIII, factor VIII; PK, pharmacokinetic; VWF:Act, von Willebrand factor activity; VWF:Ag, von Willebrand factor antigen; VWF:CB, von Willebrand factor collagen binding; VWFpp, von Willebrand factor propeptide.

Dosing will be advised by clinical pharmacologist on the basis of FVIII/FIX target trough levels as set by treating physician, in accordance to patient characteristics, previous trough levels (if a patient switches between factor concentrates), bleeding history, activity pattern, and in consultation with patient/caretakers. If desirable, physicians are also able to set FVIII/FIX target peak levels during intensive physical activities. In this way, treatment is truly customized and tailored to the needs and lifestyle of each individual as personalization is meant to be. Retrospective data of a patient, such as previous trough levels and factor levels at onset of a bleed or during sport activities, can be informative to the physician to set target levels.

Thereafter, patients will initially be on PK-guided treatment for 12 weeks. During these 12 weeks, a minimum of three factor levels will be measured and compared with predicted FVIII/FIX values to validate predicted dosing regimen ( Table 3 ). Patients on EHL will be on iterative PK-guided treatment with dose adjustment if needed based on both factor levels and bleedings. Iterative treatment is desirable in these patients as most patients initiate treatment with EHL factor concentrates after being on prophylaxis with SHL factor concentrates. Because of the lack of knowledge of most optimal (frequency and dose of) EHL factor concentrate, this period has a dose finding perspective.

For patients on SHL predicted FVIII/FIX values will be blinded to the treating physician and dosages will not be adjusted during the first 10 weeks. Thereafter, dose adjustment can be made.

A subsequent follow-up period of 24 weeks on PK-guided treatment is necessary to further collect data to establish the associations between FVIII/FIX levels and bleeding events. Only if clinically indicated, FVIII/FIX levels will be measured during bleeds ( Table 3 ). At the end of this follow-up period, one final blood sample will be taken to compare the factor level with the predicted value ( Table 3 ). Patients/caretakers will again fill in the HAL or PedHAL questionnaire and the physiotherapist will perform the HJHS to evaluate outcomes after implementation of PK-guidance.

Finally, at the end of the study, both patients/caretakers and the treating physician will fill in the VAS questionnaire, considering the experience with PK-guided dosing and EHL factor concentrate when patients have switched to an EHL factor concentrate.

Importantly, hemophilia patients, who have undergone individual PK-profiling prior to study inclusion or who have already received PK-guided treatment on SHL or EHL concentrate, are also able to participate in the study. PK profiling is required to be performed with a maximum of 1 year prior to study inclusion when <12 years of age and a maximum of 3 years prior to study inclusion when 12 years and older. Patients who already received PK guidance prior to study inclusion, will only complete the VAS questionnaire at the end of the study period, since asking the patient questions with regard to expectations on PK-guided dosing at the beginning of the study would lead to recall bias. Also, the HJHS will not be performed in these patients and PedHAL will not be completed in this subgroup as results after body weight (and bleeding) –based prophylaxis and PK-guided therapy cannot be compared.

In parallel with the prospective study, retrospective data analysis will be performed over a 12-month period prior to inclusion (if no PK profiling has been performed) or from PK profiling prior to inclusion. These data will be utilized as “real-world data” to construct and enrich available population PK models. Moreover, if patients kept a detailed patient log-on infusion dates and timing and bleeding episodes, annualized (joint) bleeding rate (A(J)BR) FVIII/FIX trough levels and FVIII/FIX levels during physical activities and at the onset of a bleed can be calculated.

Factor activity levels will be measured by local laboratories, as this reflects the real-world setting of standard clinical practice. Plasma samples are stored in case centralized measurements are deemed necessary. Laboratory specifications (assay, reagents, deficient plasma, and analyzer) applied in local laboratory will be recorded precisely. Preferably, the local laboratory assays match with the assays as used during population PK model construction.

### Bayesian Forecasting

Bayesian forecasting will be performed with the NONMEM software (Icon, Dublin, Ireland); individual PK parameters will be assessed with a limited number of blood samples. Available population PK models in literature and new models that will become available will be used. Based on the estimated individual PK parameters and the FVIII/FIX target trough and peak values as set by the physician, dosing schedules will be calculated.

#### Sample Size Calculation

In this prospective study, we aim to evaluate the predictive performance of PK-guided dosing in hemophilia patients. It is not common practice to calculate a sample size for prognostic models, and, to the best of our knowledge, it is not possible to calculate a sample size for the determination of predictive performance. What we do know is that as characteristics, such as age, body weight, activity pattern, and bleeding phenotype, are not part of the inclusion or exclusion criteria, the study population will be a reflection of the real-world and thus a heterogeneous hemophilia population. However, we aim to enroll a minimum of 50 patients in all strata together to explore the predictive performance of PK-guided dosing in real life.

### Statistical Analysis

Continuous data will be expressed as mean and standard deviation when normally distributed or median and interquartile range when not normally distributed. Categorical data will be expressed as frequency and percentage.

As described in the primary study endpoint, the predictive performance of PK-guided dosing is deemed acceptable when at least 80% of the actual FVIII/FIX levels are within ± 25% of the predicted (target) values as stated by treating professional. Both the mean error between the predicted and observed factor level and the mean absolute difference of the predicted level will be calculated. No significant bias presented as zero is included in the 95% confidence interval (CI) of the mean error. Moreover, differences between the predictive performance of different factor concentrates and age groups will be investigated and described.

Association of factor levels with bleedings will be described according to sub classifications. Comparisons of the ABR and joint status before and during PK-guidance will be analyzed using a paired *t* -test or Wilcoxon's test, depending on the distribution.

R (version 4.0.3) will be used for statistical analysis.

### Ethical Considerations

The study protocol was approved by the Medical Ethics Board of the Erasmus MC, University Medical Center Rotterdam, the Netherlands, and approved by all boards of all participating hospitals.

### Registration

The trial is registered at the Dutch Trial register with trial number: NTR7523 ( *www.trialregister.nl* ).

## Conclusion

The proposed study aims to investigate the reliability and feasibility of PK-guided prophylactic dosing of factor concentrates in hemophilia-A and -B patients in daily clinical practice. Moreover, the collected real-world data will lead to enrichment of current population PK models and an increased insight in the association of FVIII/FIX levels with bleeding episodes and daily activities.
